# An Innovative Biomedical Research Training Model: Rationale, Design, and Evaluation

**DOI:** 10.3390/ejihpe12120123

**Published:** 2022-11-28

**Authors:** Payam Sheikhattari, Shiva Mehravaran, Jummai Apata, Gillian Silver, Shamara Murphy, Sylvia Hurtado, Farin Kamangar

**Affiliations:** 1 School of Community Health & Policy, Morgan State University, Baltimore, MD 21251, USA; 2 Prevention Sciences Research Center, Morgan State University, Baltimore, MD 21251, USA; 3 Department of Biology, School of Computer, Mathematical, and Natural Sciences, Morgan State University, Baltimore, MD 21251, USA; 4 ASCEND Center for Biomedical Research, Morgan State University, Baltimore, MD 21251, USA; 5 School of Education & Information Studies, University of California, Los Angeles, CA 90095, USA

**Keywords:** research training, diversity and inclusion, undergraduate research, biomedical research

## Abstract

Much is told regarding the need for greater diversity in the biomedical research workforce in terms of race, ethnicity, and socioeconomic status. However, there are few evidence-based models that are tested and can have significant effects in this regard. Thus, there is a need for development and evaluation of innovative models that may help train a more diverse biomedical research workforce. In this study, we provided the rationale, conceptual model, and preliminary evaluation of a program called “A Student-Centered Entrepreneurship Development (ASCEND)”. This training program was designed, implemented, and evaluated between 2017 and 2020 at Morgan State University, Baltimore, Maryland, United States. The program’s conceptual model is based on four stages: Attraction and Inspiration, Ideation and Innovation, Research Implementation, and Career Growth. Results of the comparative survey between 50 students who participated in ASCEND and 86 non-member controls showed an increase in science identity, academic self-concept, science self-efficacy, and peer support. The only domain that did not show a larger increase in participants in our program compared to controls was social self-concept. In addition, a total of 59 students submitted 48 research concepts, and 16 undergraduate student projects were funded. Of participants in the Health Research Concepts Competition, 39 students graduated, and 13 were pursuing graduate programs in STEM fields at the time of evaluation. The number of research projects and trainees who started a graduate degree were also reported. The ASCEND training model fosters an entrepreneurial mindset among undergraduate students. Such a program might be effective in diversifying the biomedical research workforce. While this preliminary evaluation indicates the efficacy of the ASCEND model, there is a need for further long-term and multi-center evaluations with the trainees’ research productivity and receipt of independent funding as outcomes.

## 1. Introduction

The global preeminence of the United States in biomedical sciences as well as technology is contingent on training a large and diverse group of students to comprise the future cadre of competent and innovative scientists [1,2,3]. One of the main pillars to accomplish this strategic aim is to enroll undergraduate students from varied racial, ethnic, and socioeconomic backgrounds and properly train them in various fields of biomedicine and health sciences from their first years of higher education. In the past few decades, many interventions and training programs have tried to expose undergraduate students to research, particularly in the fields of science, technology, engineering, and mathematics (STEM). Although these programs have encouraged students to pursue scientific careers [4], they have been traditionally designed based on an apprenticeship model, where students are involved in research in a limited capacity under the mentorship of a senior scientist [5,6,7].

The apprenticeship model of research education comes with certain limitations and disadvantages. The main weakness is limited reach because these programs require a great amount of institutional and faculty resources. Students are often placed in highly technical labs under the supervision of experienced mentors [8]. Students are initially engaged in basic introductory-level assignments for a relatively long time and are gradually prepared to engage in original research. Therefore, as novices, the students are often not able to clearly understand the significance and relevance of their activities to the overall research effort. Furthermore, students who choose to engage in apprenticeship-type research often already have a sense of science identity and high levels of motivation and readiness. Lack of previous exposure to research and lack of science identity can serve as a significant barrier to racial, ethnic, and socioeconomically disadvantaged students’ participation in biomedical research, as they are often still developing a science identity and require encouragement to fully take advantage of research training opportunities. Historically disadvantaged students are often less likely to seek apprenticeship-type research experiences or may be easily bored by less-challenging and repetitive assignments. Lastly, as apprentices, students often need to follow strict standard operating procedures with limited room for creativity, ownership, leadership, and interaction with peers [9]. Many students who are originally attracted to research may gradually lose interest; only a small proportion of them will maintain the drive that is needed to continue with their pursuit of a research career. Those who succeed are mostly from racial, ethnic, and economically privileged backgrounds, often inspired by successful role models and support systems that exist in their social networks, which may not be available to racial, ethnic, and economically disadvantaged students who are traditionally underrepresented in science [1,2,3,10]. Therefore, there is a continued search for relevant evidence-based approaches for optimizing recruitment, education, and training outcomes for students from diverse backgrounds [11,12].

Federal agencies, such as the National Institutes of Health (NIH) and the National Science Foundation (NSF), have made significant investments in developing innovative research training programs [12,13]; however, most of these funded programs have followed an apprenticeship model. Incorporation of an entrepreneurial mindset into research training has the potential to overcome the limitations of the traditional apprenticeship training model by allowing students to drive the process more creatively, which can lead to greater peer support and students’ increased ownership of their research. In the real world, leading researchers have an entrepreneurial mindset: they use their creativity to generate novel research ideas, pitch their ideas to secure funding, take ownership of their projects, lead staff and other scientists, and in every step of this venture, they accept the associated risks and responsibilities. Therefore, cultivating an entrepreneurial mindset in research training has the potential to better position undergraduate researchers to answer research questions and motivate them to become highly accomplished, next-generation investigators.

### Objectives

Here, we present an educational intervention developed and implemented at Morgan State University (MSU), a Historically Black University in the Mid-Atlantic region of the United States. The overarching goal of the program, titled “A Student-Centered ENtrepreneurship Development training model to increase the diversity of the biomedical research workforce (ASCEND)”, is to foster an entrepreneurial mindset among undergraduate students from racial, ethnic, and economically disadvantaged backgrounds [14]. The ASCEND program is designed according to a central “research entrepreneurship” framework in which engaging undergraduate students in a self-directed entrepreneur-style research training program will foster their sense of autonomy, increase their level of interaction with their peers and mentors, and result in a strong sense of science identity, readiness to lead research and admission into research-oriented graduate programs [14]. While the entrepreneurship concept is typically used in business, it is used in ASCEND at MSU with the ultimate goal of incorporating critical aspects of the entrepreneurial mindset into research training.

## 2. Materials and Methods

In this paper, we first provide a general description of an evidence-based Entrepreneurial Research Training Model (ERTM) that was implemented within the context of an undergraduate student organization, the ASCEND Student Research Center (SRC). Then we present a detailed description of our initiative mapped to the four stages of the ERTM. Then, we present a summary of the evaluation results. Finally, we discuss their relevance to undergraduate research training.

### 2.1. ASCEND’s ERTM

ASCEND is a program funded by the NIH BUILD initiative [https://nigms.nih.gov/training/dpc/Pages/build.aspx accessed on 1 September 2022], which is one of three initiatives of the Diversity Program Consortium (DPC, https://nigms.nih.gov/training/dpc accessed on 1 September 2022). The goal of the DPC is to enhance the diversity of the NIH-funded workforce. The BUILD initiative consists of ten awards to undergraduate institutions that serve geographically and racially diverse populations and include historically Black colleges and universities, Hispanic-serving institutions, Asian-American/Native-American/Pacific-Islander-serving institutions, and projects with targeted outreach to special populations. Given that Morgan State is an HBCU, most students are from minority backgrounds and/or are Pell-grant eligible.

**Figure 1:**
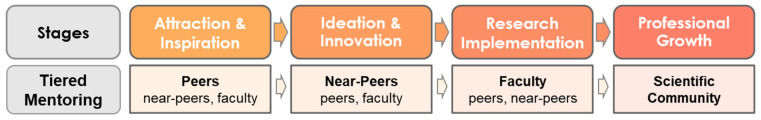
The four stages of the ASCEND Entrepreneurial Research Training Model and its alignment with the tiered mentoring program.

The ERTM was ASCEND’s foundational, evidence-based model for nurturing an entrepreneurial research mindset through scaffolding, peer support, and motivation to engage in a student organization dedicated to building a culture of undergraduate research [15,16,17,18,19,20,21,22,23]. This model was developed as a step-by-step approach to scaffold the growth of undergraduate students toward assuming leadership roles in research. As depicted in Figure 1, the ERTM consists of four main stages: Attraction and Inspiration, Ideation and Innovation, Research Implementation, and Professional Growth. At each stage, students engage in various activities and acquire certain skills that prepare them for the next stage. While activities may be specific to a given intervention, in general, students start with less-challenging activities that can be fun and engaging and gradually progress toward more sophisticated tasks and responsibilities. At later stages, students receive more specialized training and support, and they engage with their respective scientific communities. To have an authentic entrepreneurial research experience, students are given full ownership of their co-curricular activities and research projects; they network, are inspired, express their creativity, and impart their dreams and ambitions with their peers, near-peers, and faculty mentors (tiered mentoring); and collaborate on projects from the start by proposing their own research topic, developing research methods, writing proposals for small grants, and moving the project forward as a team. Undergraduate students with prior research experience play a critical role in attracting and inspiring their peers. Through interaction with their peers, students can ask their initial basic questions in a safe and friendly environment and learn more about the benefits of conducting health research. Near-peer mentors (NPMs), who are masters and doctoral students, will further advance the students’ science identity by helping them with ideation and innovation. Students assist their undergraduate peers with finding relevant resources, engaging in available learning opportunities, revising their concepts, and connecting with potential faculty mentors (see Section 2.4). This tiered model of student mentoring may have the potential to more effectively prepare students to work with their future faculty mentors, which is different than the typical teaching-assistant role of the near-peer under apprenticeship-style research. Outcomes were assessed at various stages and ranged from psychosocial factors that have an impact on persistence in STEM to concrete scientific writing and analytical competencies. The ultimate goal was to increase the quantity and quality of students who pursue graduate biomedical training.

### 2.2. Aims and Goals of the Program

The main goal of the program was to support and guide student members through the four stages of ASCEND’s ERTM (Figure 1). A faculty advisor and a coordinator were responsible for overseeing the progression of the students through the four stages shown in the figure. These activities are intended to ignite undergraduate students’ interest in health research, expand members’ support groups and networks (including peer and near-peer researchers, scientists at MSU and partner institutions, and key contacts at their prospective graduate schools), provide them with and/or guide them to available training and resources, and help them to achieve the full benefits of engaging in authentic research experiences, including better preparedness for graduate school and enhanced likelihood of pursuing a career in health-related and biomedical research.

### 2.3. Tailored Design

**Figure 2:**
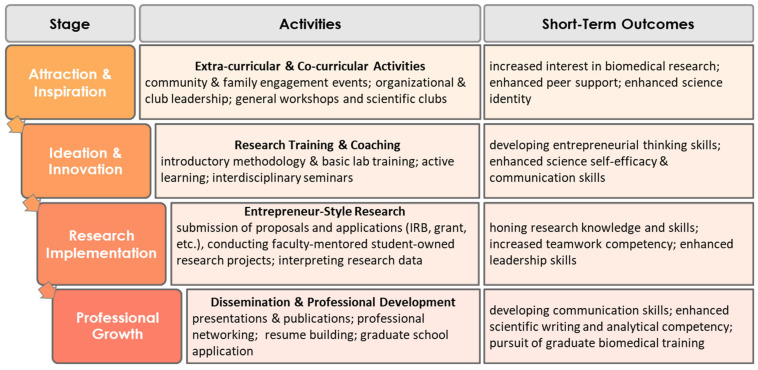
The ASCEND Entrepreneurial Research Training Model and the activities supported by the Student Research Center.

The students who entered the ERTM, however, varied in background and previous experiences. Students progressed through the stages at a pace that was tailored to their experience. They participated in relevant research activities to meet their own needs and help them build the necessary skills that prepare them for the next stage. In addition to motivation and hard work, students’ success relied on the tiered mentoring support system that was embedded in the model. This means that as students made progress, not only were they rewarded with more opportunities and resources for their own success, but they could also contribute to the progress of less-active members and those junior to them as tutors, peer mentors, and role models. In the next sections, we provide more detailed descriptions of the activities of each ERTM stage. As the aim was to train biomedical researchers, all stages of our program had an emphasis on developing a “biomedical” mindset (Figure 2).

#### 2.3.1. Stage I: Attraction and Inspiration

The first stage was to inspire students from racial, ethnic, and economically disadvantaged backgrounds to conduct biomedical and health-related research by attracting them to and recruiting them into the SRC, a relaxed environment where they could meet like-minded peers and learn from more senior researchers. Students from any major or classification were welcome to join the research education program as long as they were interested in engaging in biomedical and health-related research. As newcomers gathered in the SRC lounge area, they were welcomed by their peers who were slightly more experienced and supported their gradual orientation into the next levels. New members were also inspired by those who had already made accomplishments; the new members were surrounded by a “community of researchers” as role models.

In terms of activities, members mostly engaged in introductory extra-curricular and co-curricular events that helped them become acquainted with the organization and foster their interests. They network with peers and provide input about the services, programs, and initiatives they need or desire. Students were encouraged to recruit new members, join a committee, participate in workshops and site visits, and organize or volunteer in community-oriented events and research. These experiences allowed them to gain a sense of belonging and start sharing their ideas with like-minded peers, working as a team, learning critical thinking and communication skills, and discovering their interests, potential, and professional goals. Once students had identified their research topic of interest, they were placed in touch with peers and faculty who were working on similar, relevant research topics and projects to spark the students’ creativity and encourage them to start thinking about their own research. We hypothesized that at the end of this stage, students would have a stronger sense of peer support in science and research, an increased sense of science identity, and greater motivation to conduct research. Some students may start developing ideas of where to find topics and resources for conducting research.

#### 2.3.2. Stage II: Ideation and Innovation

Students were encouraged and empowered to think outside the box. This was designed as the beginning of their entrepreneur-style research endeavor. The success of such an initiative required high levels of creativity, passion, and participation in thought-provoking activities. To incentivize the process, the SRC Executive Board (E-Board) established a “Member Incentive Program” as both a reward system to enhance members’ engagement, productivity, and creativity as well as an accountability measure for ensuring fair resource allocation. As part of this program, members receive points based on their participation and contributions in the areas of community and university services, commitment to leadership roles, and accomplishments in research and academic performance. These points were used toward achieving higher SRC membership levels (Bronze, Silver, Gold, and Diamond), which entitle members to more benefits, including increased access to facilities, access to advanced training, and financial support for professional development (e.g., travel to professional conferences) and graduate school preparation activities.

Members were expected to work on their own research ideas and develop original research projects. As they engaged more actively, they continued to be introduced to peers with shared interests during general body and committee meetings, planning retreats, and training workshops. At this stage of the ERTM, students may have developed a general sense about their area of research (e.g., investigation of the biological basis of schizophrenia) but may not have yet developed a clear research question. It was necessary to guide them into the practical next steps and facilitate their access to experts and resources. To transform preliminary ideas into feasible researchable concepts, students were advised, by their near-peer mentors and the faculty, to take introductory-level research methods courses and participate in research workshops, reach out to and interact with potential faculty mentors, and participate in interdisciplinary seminars. Students were also encouraged to work in groups, so they could practice their team-building and leadership skills. By the end of this stage, each member had formulated a well-defined research question and had established contact with at least one faculty member with relevant research background and expertise. By the end of stage two, students were expected to have enhanced knowledge of research, science self-efficacy, critical thinking skills, and leadership skills.

#### 2.3.3. Stage III: Research Implementation

The main activity in this stage was engagement in research implementation. This could be participation in individual or team-based entrepreneur-style research studies or working on other faculty-mentored research projects. In addition, students received notifications and announcements about internal and external research scholarships and internship opportunities, and they were strongly encouraged to apply.

A Health Research Concepts Competition (HRCC) initiative was designed to help MSU undergraduate students develop and execute their own research projects, which could take about one or two academic years. Students interested in entrepreneur-style research received support to identify a research mentor (see Section 2.4), so they could further develop their research concepts into proposals, apply for and obtain funding, conduct their research, analyze their data, interpret their findings, and present/publish their results.

**Figure 3:**
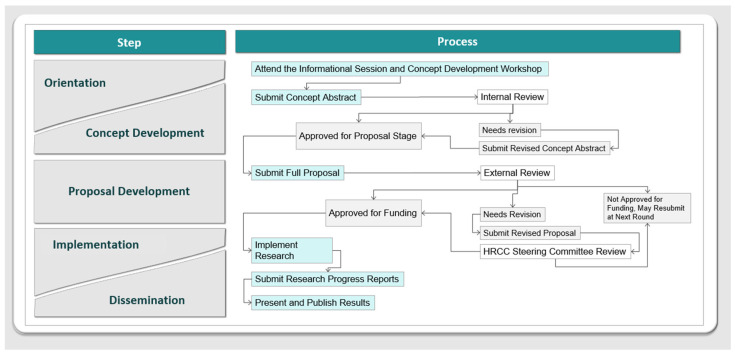
The phases and processes of the Health Research Concepts Competition of the ASCEND Student Research Center.

The HRCC was an initiative for this stage with phases and processes that mimic the real-world grant process. While rigor in research was emphasized, broadening the horizons of science was not critical. Therefore, this was more a “practice round” of rigorous research rather than research intended to be published in high-impact journals. Through the HRCC, students first shared their research ideas with faculty and peer mentors and shaped such ideas into a 600-word summary, which was then reviewed and endorsed by a faculty member. The original student investigator served as the principal investigator, and the endorsing faculty member served as the lead mentor who guided the student through each step of the research process (Figure 3). s Submitted concepts were reviewed internally by volunteer faculty members and staff, and students were provided with feedback to revise their research summary. This internal review focused on structure and basics such as scientific writing, statistics, citations, and literature review. Students with meritorious concepts received support to develop their research summary into a detailed research proposal. Submitted proposals were then reviewed and scored by at least two senior faculty investigators from research-intensive institutions with expertise in the respective field. External review was conducted by individuals with expertise on the subject, who were able to provide more nuanced feedback. Student investigators and their mentors continued working on their proposals and revised them according to the external reviewers’ comments. They also delineated the mentoring and research tasks for conducting the proposed research; this allowed students to define roles for potential collaborators, invite others to join the team as needed, and build teams based on expected outcomes and the scope of the work. Funded projects proceeded to the implementation phase, for which they submitted Institutional Review Board or Institutional Animal Care and Use Committee applications to MSU (as applicable) and research progress reports.

Students were granted access to advanced research and lab training relevant to their proposed study at MSU or specialized labs at partner institutions. The expected outcome for successfully mastering stage III was completing an undergraduate research project suitable for scientific presentation and peer-reviewed publication. By the end of this stage, we expected an increase in knowledge of research procedures, science communication skills, research self-efficacy, and teamwork skills of the participants. Further, we expected the students to have a stronger connection with faculty.

#### 2.3.4. Stage IV: Professional Growth

By this point, students were ready for meaningful engagement with the larger scientific community. Those who had successfully completed their research projects received additional support to present their findings at research conferences and publish them in peer-reviewed journals. Such experiences subjected them to critical reviews like what faculty scientists often experience. Further, they were urged to engage in robust networking with students and faculty within and outside the primary institution.

To enhance student preparedness for graduate school, they were provided with or directed to opportunities for student-centered academic and career development, research, and mentorship training. Graduate school preparation opportunities included (1) workshops, training, and tutoring sessions on mathematics and scientific writing, psychosocial skills, bench science techniques, and novel technologies; (2) student-centered initiatives and scientific sessions such as SWAGs and journal clubs; (3) application preparation activities (mock interviews, GRE prep, curriculum vitae preparation, etc.); (4) the weekly Interdisciplinary Seminar Series that introduces students to biomedical scientists representing a wide range of disciplines (psychology, chemistry, biology, mathematics, social science, environmental science, and engineering); and (5) annual research events such as leadership and strategic retreats, research and academic fairs and exhibitions, and the annual Undergraduate Research Symposium.

In addition, students were also provided with a list of online resources that facilitates and encourages self-guided and self-paced instruction that was expected to further enhance students’ communication, scientific writing, and analytical skills as well as prepare them for graduate biomedical training. These resources included but were not limited to Smarthinking, an online tutoring service available to MSU students, and Khan Academy, an educational service that includes online interactive challenges, assessments, and videos on a variety of academic topics. By the end of this stage, we expected students to be ready for graduate studies and to have formed a clear and strong opinion of whether to pursue a biomedical research career.

We recruited individuals from different disciplines if they were willing to do biomedical and health sciences research. Consequently, we have students from non-biomedical majors, but all conducted and learned biomedical science work. Our emphasis on a “biomedical” mindset was made possible because our ads, lectures, workshops, projects, faculty, and mentors were all from biomedical and health sciences research. As all our materials from educations, lectures, and mentors were specifically focused on “biomedical sciences”, the result is the stimulation of a “health and biomedical mindset”.

### 2.4. Tiered Mentoring According to the ERTM

As depicted in the model (Figure 1), students’ success in navigating the stages of the ERTM relied on the support of various mentors and a tiered mentoring program. This structured peer and faculty mentoring process has been shown to be effective in preparing racial, ethnic, and economically disadvantaged students and fostering more inclusive educational environments [24,25]. As such, mentorship was seen as an ongoing process for members of the program throughout all stages, and as they made progress to higher stages, they, in turn, served as mentors for those junior to them. In stages I and II, peer and near-peer mentors (especially SRC members in advanced stages of the ERTM) had a major role in helping new members build their confidence and inspiring them to ideate and develop research concepts. As students acquired the requisite skills and become more prepared to conduct their own research, they searched for faculty mentors that best matched their research interests from among a pool of faculty members who have expressed interest in serving as mentors. A repository of potential faculty mentors was maintained on the program website. To facilitate mentor selection, a mentor-mentee mixer was held so that students could meet potential mentors in an informal and relaxed setting. This led to building a mentoring relationship and rapport over time between students and faculty with shared interests. Students and their mentors engaged in preparing individual development plans and were invited to participate in training events modeled after the *Entering Mentoring* training curricula developed by the National Research Mentoring Network [26].

At the implementation stage, near-peer mentors initially had a more critical role than faculty mentors in helping undergraduate students develop their research proposals and conduct their studies. Near-peer mentors were often graduate students from the same field of science as the undergraduate student, with more research experience and knowledge about lab procedures. Some benefits of undergraduate students engaging near-peer mentors were: (1) the undergraduate students were more comfortable asking the near-peer mentors basic questions, most (if not all) of which they can answer; (2) near-peer mentors were usually more accessible than faculty mentors, and (3) faculty mentors could be more effective in their role when the undergraduate students are prepared and supported by their near-peer mentors. As the students gradually progressed in their research, they needed more specialized training and individually tailored mentoring experience. Hence, members of specialized teams with identified mentors were encouraged to initiate a self-assessment of undergraduate students’ short-term and long-term goals, including current skills they possess and skills they would like to develop. This was then written into an Individual Development Plan (IDP) that included research goals, suggested coursework, skills development (e.g., improving verbal and written communication, leadership, and analytical skills), teamwork, and community outreach activities. The IDPs were prepared in collaboration with the students’ advisors, shared with their research mentors and the SRC coordinator, and maintained as an electronic document that captures their participation in and progression through the program. IDPs documented all learning objectives and training activities needed to facilitate their attainment, as well as timelines of activities and expected hallmarks of success developed by the NIH [27]. Creating IDPs has been recommended by the National Academies of Science as an important tool for tracking and enhancing students’ core competencies [1,2,3].

Mentees and mentors were given separate orientations and structured training to clarify expectations, improve communication skills, reduce any potential implicit biases, and prepare them for ethical and professional interactions. Faculty mentors could expect incentives such as release time, lab supplies, travel grants for presentations at scientific conferences, etc. However, the main motivation for senior scientists and junior protégés to work together would be the collaboration on mutually beneficial research activities and products. At stage IV, students were growing and transforming into more competent researchers through interaction with the scientific community, which meant they were growing their own professional network to further enhance their research competencies and prepare for graduate-level training in biomedical sciences.

## 3. Results

### 3.1. Preliminary Evaluation

As illustrated in Figure 2 and mentioned in the description of the ERTM, specific outcomes are expected at each stage. These include psychosocial changes such as an increased sense of peer support, science identity, and science self-efficacy, as well as metrics ranging from the number of students attracted (stage I) to the number of students accepted into graduate school (stage IV). These outcomes were tracked and documented using a comprehensive database that was established to satisfy the requirements set forth by MSU’s Office of Student Life and Development (OSLD) for undergraduate student organizations, the NIH as the funding agency, and the evaluation undertakings of ASCEND, which implements a mixed methodology to study program outcomes longitudinally.

### 3.2. ASCEND Members

As a result of this program, the SRC was officially registered as an undergraduate student organization in 2016 and transitioned to be part of a newly established Office of Undergraduate Research in the fall 2021. Data reported here cover this period. Within this timeframe, the SRC continuously attracted members interested in health-related research; the SRC recruited close to 250 members. As summarized in Table 1, in 2020, SRC active members have been from many majors and all classifications. Most participants were recruited from the department of biology.

### 3.3. Psychosocial Factors

To evaluate the effectiveness of the SRC in enhancing psychosocial factors, we have developed a multi-section retrospective pre-post questionnaire (the points in time being one year ago and currently) using 30 items from previously administered surveys [28]. The first part of the questionnaire collects students’ demographics, academic information (major, classification, GPA, and enrollment status), membership in student organizations, and receipt of any student training scholarships. The second part asks them about engagement in research-related activities during the summer or academic year. The remainder of the questionnaire includes the following five constructs: (1) science identity, (2) academic self-concept, (3) personal and social self-concept, (4) science self-efficacy, and (5) peer support for research and science (see Appendix A for more details).

In the most recent round, the survey was administered to SRC members as well as to students in upper-level biology courses and attendees of the MSU Graduate and Professional Careers Conference as our comparison group. A total of 256 students completed the survey. Of these, we excluded freshmen, graduate students, those with a GPA less than 2.8 (the minimum required for SRC membership), and students who were in student research training scholarship programs, including ASCEND Scholars (ASCEND scholars were excluded from the comparison group). Of the remaining 136 students, 50 were SRC members (41 female and 9 male), and 86 (70 female and 16 male) were included in the analyses as the comparison group. These two groups were compared in terms of one-year change in the five domains of interest using multivariate analysis with adjustments for gender, classification, and GPA. As mentioned, most of our participants were female. This is in line with the distribution of gender in statistics at minority-serving institutions and specifically at MSU. As our numbers show, in our university and departments, women are overrepresented, and thus our sample is disproportionately female. The percentage of women in ASCEND is similar to the percentage of women in departments such as biology and psychology, which were most active in recruitment.

The results of the survey are summarized in Table 2. In all domains except social self-concept, there was a significantly greater increase in the measures over the past year in SRC members than in the comparison group, such that, although mean pre-test scores were often initially lower for SRC members, mean post-test scores were significantly higher.

### 3.4. Qualitative Results

SRC members who participated in focus group discussions were of the belief that their positive changes, accomplishments, and aspirations were due to their participation in the SRC. They stated that the SRC is a place that has a welcoming environment where they feel a sense of belonging and form strong bonds with like-minded students; where they can engage in activities that improve their social, academic, leadership, and research skills; where they can get help to set up a clear direction for their future and find inspiration to aim higher in their career paths; where they have the opportunity for networking with students and faculty at other Historically Black Colleges and Universities (HBCUs) and minority-serving institutions through conferences and workshops; and where they can participate in workshops that help them prepare for successful graduate school applications. When asked about challenges and needs, most SRC students stated that they were extremely satisfied with their SRC experience and they would not change anything about it. Sample quotes are presented in Table 3.

### 3.5. The Health Research Concepts Competition (HRCC)

The HRCC has generated enthusiasm and attracted many students and faculty. As presented in Table 4, between 2017 and Fall 2020, a total of 59 students have submitted 48 research concepts, and 16 undergraduate student projects have been funded for USD 5000 each. Of these HRCC participants, 39 students have graduated, and 13 are now pursuing graduate programs in STEM fields.

## 4. Discussion

ASCEND’s ERTM is a novel student-centered model designed to enhance the quality of undergraduate research training and prepare students from backgrounds that are underrepresented in the biomedical research workforce for a successful and productive research career. ASCEND’s ERTM is designed to systematically help learners enhance their capabilities through peer and faculty support. In this model, students are the main drivers of their own learning experiences scaffolded by peers, instructors, and mentors. This concept was originally defined by Vygotsky in the Zone of Proximal Development (ZPD) theory. Vygotsky defines the Zone of Proximal Development as the distance between the actual versus potential developmental levels of learners with or without scaffolding that comes in stages, which mirror the four stages of the ERTM [29]. More specifically, stages one and two of ASCEND’s ERTM provide a pathway from expert- to self-assistance, while in stages three and four, concepts are internalized, and learners become more independent by going through an iterative process and interacting with the scientific community [30]. In this paper, we compared select psychosocial outcomes from participants in ASCEND’s SRC with a comparison group of non-SRC participants. Our findings show that the model is educationally sound and feasible. The SRC has recruited about 250 undergraduate student members interested in health research from almost every major and classification, which indicates the success of the program in attracting and expanding science training to undergraduate students with various interests.

The initial results indicate that the SRC’s programs and activities have been significantly effective in helping students enhance their levels of peer support, science identity, academic self-concept, and science self-efficacy compared to control groups. Several previous studies have shown that students with positive perceptions about themselves as a scientist and their confidence in their ability to excel academically are important predictive factors of their future success [31]. Altermatt [15] wrote that peer support can be a powerful source of inspiration and a predictor of students’ future academic success, mainly through validating good ideas and supporting actions.

The stages of the ERTM mimic the real world in at least three ways. First, they may improve students’ metacognition by enhancing awareness of their own passions and thought processes [20]. Second, they provide—with scaffolding—learning that starts with easier levels and gradually advances to more difficult ones. Students often lose interest when their first exposure to research is perceived as being too difficult [21]. Through reflective and project-based learning, students examine their issues of interest and learn as they develop their future research projects [16,32]. In the ERTM, newcomers go through a gradual orientation, starting first by meeting like-minded peers with slightly more experience to create a more pleasant, welcoming, and relaxing the first impression. Qualitative assessment has shown that most students found the SRC space to be like a home with plenty of peers ready to help them. Peer mentoring at the entry level is a powerful strategy to better acquaint research trainees with the environment [19]. After the orientation, the role of near-peer mentors and graduate students becomes more prominent in guiding the students, providing basic training, and facilitating students’ connections to more-senior researchers. This tiered mentoring system has the potential to scaffold students’ reflections leading to higher levels of introspection, self-analysis, and open-mindedness that have been reported to be effective in enhancing the quality of the training and research productivity [33,34]. According to Gilbert and Trudel [18], peers learn how to coach and mentor others, often through reflections in different stages of learning and by engaging with the subject and their peers. Third, ASCEND’s ERTM is based on a student-centered approach where the students are empowered drivers of change; they select their topic and choose their mentors. Student-centered learning is reported to lead to more creativity, self-reliance, and future growth by providing the students with the opportunity to control and guide their own learning process while in training [35]. The combination of the student-centered approach and engagement in the SRC creates ample opportunities for appreciative inquiries between students and with their mentors, which is a strength-based strategy built upon students’ success that has been reported to enhance both students’ scientific competencies and leadership skills [36]. The results indicate that the SRC has served as a natural bridge and gateway to other opportunities by preparing students for more advanced scholarship opportunities, external internship programs, and graduate schools.

The entrepreneurial mindset is about having ideas and taking risks in realizing them; such a mindset requires creativity and determination [37]. The Member Incentive Program is designed to incentivize and nurture creativity, research entrepreneurship, and agency, which has been effective in creating a more accountable system and positive pressure for change. In addition, pilot grant programs, even at small dollar amounts, have been quite effective incentives for junior researchers [38,39]. Through the HRCC program, undergraduate students can submit their concepts and proposals for funding as principal investigators. This has been a powerful opportunity and motivation for the students to act like entrepreneurs and work with their peers and faculty mentors to ideate and further develop their own research. Furthermore, the ERTM has the potential to broaden the horizons of young scientists who may mainly consider academic research careers while ignoring other opportunities as independent researchers and entrepreneurs in start-ups, private industries, or foundations.

The SRC, as a student club, has been an important venue for finding peer support and practicing leadership to govern the organization. Peers who share similar backgrounds have functioned as role models for discovering what might or might not work in terms of research [40]. Elements of this model could be utilized to foster a culture of research entrepreneurship and maximize the impact of traditional research training approaches by inspiring a better sense of self-confidence and efficacy, improving the understanding of complex subjects, fostering a sense of science identity, increasing creativity, and preparing students for more effective engagement and interaction with their senior mentors. Another set of issues affecting the future of the biomedical workforce concerns the nature of the training young scientists receive and the mismatch between that training and their career prospects. This mismatch might be addressed by providing training in the context of a student organization where members enjoy connecting with their peers regardless of their disciplinary background and take a more active role in their own learning. The ERTM is relevant to all students. However, racial, ethnic, and economically disadvantaged students may especially benefit from the model as they are less likely to have been exposed to ideas, role models, and opportunities compared to their “majority” peers.

The ERTM provides a simple, replicable, and feasible model to produce self-directed resilient learners who are unafraid to take risks, engage in real research, and learn from their mistakes. This contrasts with traditional apprenticeship models, which do not scaffold learning or encourage students to own the production of knowledge, where students learn from experts in environments with less opportunity for peer support and autonomy. Furthermore, the SRC fits well within the mission of almost all undergraduate programs to advance learning and leadership. Having an academic peer (student) organization is an important capacity for recruiting more members, raising funds, creating leadership opportunities, and planning needed activities and training. Some potential institutional benefits include but are not limited to enhancing the quality of research training, creating more peer support, enhancing the diversity and retention of undergraduate students in research programs, and increasing the research productivity of the institution. According to Hurtado et al., joining a pre-professional or departmental club during the first year of college significantly increases racial, ethnic, and economically disadvantaged students’ likelihood of staying in their major. They suggest that membership in such organizations may provide students with opportunities to engage with peer groups that share similar interests and professional goals, which can help reinforce their science identity and subsequent professional endeavors [41]. Other studies also have reported on the positive effects of participation in student organizations among Latinos and African Americans [42,43,44]. The upfront costs for creating a student research center are fairly minimal, with great potential for return on investment. Further, the ERTM has the potential to substantially enhance the effectiveness of current undergraduate research programs by more actively involving students in driving the process. Therefore, the SRC’s relatively small cost can be justified, and the program has a reasonable likelihood of being implemented and sustained.

### Limitations

The educational philosophy of scaffolding behind the stages of the ERTM makes it easier to fit with the mission of most undergraduate colleges and universities with an explicit focus on undergraduate research. So far, this model has been tested within the context of an HBCU since 2016. Therefore, the long-term impact of the program cannot be established yet, and our sample size is relatively small. There is a need for larger sample sizes, longer follow-ups, and testing of the model under varied conditions. Student-focused projects in many elite campuses, such as student theses and capstone projects, are a requirement for graduation. However, some experts have expressed skepticism about the feasibility of such an approach in under-resourced settings and with racial, ethnic, and economically disadvantaged students. This concern is founded mainly on the assumption that racial, ethnic, and economically disadvantaged students may not know enough to formulate their research questions and design their own studies. However, even though the ERTM has not yet been tested in other similar settings, the initial feasibility of the model in the context of an HBCU could be a plausible indication that with scaffolding and tiered mentorship, this approach may be successful with other students who have not had significant research experience. Despite these limitations, the ERTM and the SRC are novel ideas with promising early results, using both qualitative and quantitative data and measuring psychosocial impact with reliable instruments. Another limitation was the lack of measurement of the IDP process. Further, other universities and institutions should tailor the program to their demographic and educational background, needs and resources. For example, at MSU, many students in the program were enrolled from the biology department, and participants were disproportionately female. These conditions may vary at other institutions, and such changes may have implications for implementation and evaluation of the program. Thus, we do not imply that our results would replicate across all other settings. The Cronbach’s Alpha discrepancy between pre-test and post-test was also another limitation to examine in the future. At MSU and beyond, we are continuing to study and will report on the model in a few years; we welcome collaborations with other institutions.

We also need to explain the variation in numbers (sample size) across tables. Since its establishment, the SRC recruited over 250 undergraduate students as “SRC members” (stated under 3.2). This is an aggregate number, but there was considerable variation in the number of active members across semesters. As new students join the program, many students would graduate. For example, in spring 2020, the SRC had 74 active student members. When surveys were administered, only active members were invited to participate, and from this number, only a proportion participated in the survey. We have mentioned in the results that 50 SRC members responded to the survey. Table 1 breaks down the distribution of the 74 students who were SRC members in spring 2022. Table 2 summarizes the results of the survey that were only returned by 50 SRC members. Table 3 presents the number of SRC students who participated in the HRCC at some point between spring 2017 and spring 2020 (*n* = 59).

## 5. Conclusions

ASCEND’s ERTM is a promising model with initial evidence for its appeal among racial, ethnic, and economically disadvantaged students and its feasibility within the context of institutions of higher education. The program has the potential to enhance students’ autonomy and ownership of the research training process. This model may be particularly appropriate for minority–majority institutions and universities with limited resources. Some evidence suggests that implementing the model within the context of a student-led organization may enhance the quality of research training, possibly due to the higher likelihood of peer support, diversity, interdisciplinary teamwork, and student governance inherent to the ERTM. More research is needed on evaluation of this innovative training model across various settings.

## Figures and Tables

**Table 1:** Major and classification distribution of ASCEND Student Research Center active members in 2020.

Major	Classification as of Spring 2020				Total
	Freshman	Sophomore	Junior	Senior	
**Biology**	2	7	13	18	**40**
**Chemistry**			1	2	**3**
**Psychology**		1	3	5	**9**
**Nursing**		1	2		**3**
**Other**		7	4	8	**19**
**Total**	**2**	**16**	**23**	**33**	**74**

**Table 2:** Results of the comparative survey between 50 ASCEND Student Research Center (SRC) members and a comparison group of 86 non-member controls. Pre and post numbers represent mean scores on a 5-point Likert scale for domains number 1 to 4 and the average number of friends for domain number 5.

*Domain*	*SRC Members (n = 50)*			*Comparison Group (n = 86)*			p-Value *
	*Pre*	*Post*	*Change*	*Pre*	*Post*	*Change*	
***Science Identity***	3.31	4.18	0.87	3.4	3.76	0.36	0.027
***Academic Self-Concept***	3.68	4.22	0.54	3.75	4.01	0.26	0.005
***Social Self-Concept***	3.52	4.20	0.68	3.79	4.23	0.44	0.483
***Science Self-Efficacy***	3.05	4.05	1.00	3.27	3.74	0.47	0.029
***Peer Support***	3.35	6.43	3.08	4.50	5.50	1.00	0.002

* Inter-group comparison of 1-year change, adjusted for gender, classification, and GPA.

**Table 3:** Sample student quotes extracted from focus group discussion transcripts.

*Theme*	*Sample Quote*
***Positive Changes***	“[The] SRC makes us better and we make each other better.”
***Social Skills***	“For me ASCEND and the SRC really changed my life. Before the SRC, I wasn’t as sociable as I am now.”
***Peer Support***	“You just really build great relationships with the people there [the SRC].”
***Academic Aspirations***	“I wasn’t putting in effective study hours, but now that we have a facility like this, and I saw this, it made me want to study and do my work. It made me want to put my best foot forward in all my classes.”
***Research Competency***	“It provides us an opportunity to be an independent researcher.”
***Leadership Skills***	“It’s improved my leadership skills and professionalism.”
***Networking***	“Not only did [the SRC] give me an opportunity to go to the Annual Biomedical Research [Conference for Minority Students] but that also helped me network with a wide variety of schools for getting a PhD.” “Now it has allowed me to not only connect with the people in the SRC but also students in other organizations. So now that is a gateway [to] just more networking opportunities.”

**Table 4:** The Health Research Concepts Competition: Summary of the number of concepts and funded projects, spring 2017–spring 2020.

*Metric*	*Number*
***Student participants***	59
***Concepts submitted***	48
***Proposals reviewed***	21
***Projects funded***	16
***Projects completed***	5
***Conference presentations***	7
***Journal publications***	1
***Students graduated***	39
***Students pursuing graduate programs***	13

## Data Availability

The deidentified data sets used in the current study are available from the corresponding author upon reasonable request.

## Appendix A

Overview of the five constructs in the questionnaire developed for measuring changes in psychosocial factors in undergraduate students at Morgan State University.

(1) Science Identity

The 7 items in the science identity construct use a 5-point Likert scale to ask about the “level of importance” of (1) becoming an authority in a scientific field, (2) obtaining recognition from colleagues for scientific contributions, (3) making a theoretical contribution to science, and the “level of agreement” with statements about (4) having a strong sense of belonging to the community of biomedical scientists, (5) deriving personal satisfaction from contributing to a team that is doing important research, (6) thinking of oneself as a biomedical student, and (7) feeling like one belongs in the field of science. The Cronbach’s Alpha coefficient of this scale is 0.84 for pre-test items and 0.90 for post-test items.

(2) Academic Self-Concept

For this construct, students are asked to rate themselves on certain traits compared with the average person their age on a 5-point Likert (1 = lowest 10%, 2 = below average, 3 = average, 4 = above average, and 5 = highest 10%). The traits include (1) academic ability, (2) drive to achieve, (3) mathematical ability, and (4) intellectual self-confidence. The Cronbach’s Alpha coefficient of this scale is 0.79 for pre-test items and 0.74 for post-test items.

(3) Personal and Social Self-Concept

For this construct, students are asked to rate themselves on certain traits compared with the average person their age on a 5-point Likert similar to that for the academic self-concept. The traits are (1) competitiveness, (2) cooperativeness, (3) creativity, (4) leadership ability, (5) public speaking ability, and (6) social self-confidence. The Cronbach’s Alpha coefficient of this scale is 0.82 for pre-test items and 0.39 for post-test items.

(4) Science Self-Efficacy

This domain includes 6 items examining students’ research self-efficacy by asking “how confident” they felt about performing various research-related tasks. Responses were recorded on a 5-point Likert scale from 1 = not at all, 2 = somewhat, 3 = moderately, 4 = very, and 5 = absolutely. The tasks are (1) using technical science skills (use of tools, instruments, and/or techniques), (2) generating a research question, (3) determining how to collect appropriate data, (4) explaining the results of a study, (5) using scientific literature to guide research, and 6) integrating results from multiple studies. The Cronbach’s Alpha coefficient of this scale is 0.94 for pre-test items and 0.93 for post-test items.

(5) Peer Support for Research and Science

The peer-support section comprises 7 items asking about the number of friends or peers (1) who can help them if they have a question about their research, (2) who are ready to work with them on their research, (3) whom they help with their research, (4) who encourage them to do research, (5) who encourage them to apply to graduate school, (6) whom they encourage to engage in research, and (7) who have the same goal of getting into graduate school and becoming researchers. The Cronbach’s Alpha coefficient of the scale is 0.89 and 0.90 for pre-and post- tests, respectively.
